# Leptin regulation of neuronal excitability and cognitive function

**DOI:** 10.1016/j.coph.2007.10.006

**Published:** 2007-12

**Authors:** Jenni Harvey

**Affiliations:** Neurosciences Institute, Ninewells Hospital and Medical School, University of Dundee, Dundee DD1 9SY, United Kingdom

## Abstract

Leptin, a hormone produced by adipocytes, provides signals to specific regions of the hypothalamus to control energy homeostasis. However, the past decade of research has not only revealed that leptin receptors are widely expressed in the CNS, but has also identified numerous additional functions for this hormone in the brain. In particular, there is evidence that leptin influences neuronal excitability via the activation as well as trafficking of specific potassium channels in several brain regions. Leptin-induced alterations in neuronal excitability have been implicated in the regulation of food intake, reward behaviour and anti-convulsant effects. A number of studies have also identified a role for leptin in cognitive processes that involve activation of leptin receptors in limbic structures, such as the hippocampus. Indeed, leptin influences hippocampal-dependent learning and memory, and more recently leptin has been shown to have anti-depressant properties. Characterisation of these novel actions of leptin is providing valuable insights into the role of this hormone in the regulation of diverse neuronal functions in health and disease.

## Introduction

It is well established that the adipocyte-derived hormone leptin plays a fundamental role in the regulation of food intake and body weight; actions mediated by the activation of leptin receptors expressed on specific hypothalamic nuclei. However, leptin receptors are also widely expressed in many extra-hypothalamic brain regions including the cortex, hippocampus, brain stem and cerebellum. Moreover, a growing body of evidence indicates that leptin is a pleiotropic hormone that has diverse actions throughout the CNS. Here, the recent advances in leptin neurobiology are reviewed, with particular emphasis on the role of this hormone in regulating neuronal excitability and cognitive function.

## Leptin and its receptors

Leptin is the 16 kDa protein product of the obese (*ob*) gene that is predominantly made and secreted by white adipose tissue and circulates in the plasma at levels relative to body fat content [1,2]. In addition to acting on numerous peripheral tissues, leptin can enter the brain via saturable receptor-mediated transport across the blood brain barrier [3] or via the cerebrospinal fluid [4]. Leptin reaches most regions of the brain via the former transport process [5]. Leptin may also be made and released locally in the CNS as leptin mRNA and protein have been detected in some brain regions [6].

Initially the leptin receptor (Ob-R), which is encoded by the diabetes (*db*) gene, was isolated from mouse choroid plexus [7]. Alternate splicing of the *db* gene generates multiple variants of Ob-R mRNA and at least six receptor isoforms (Ob-Ra-f) have been identified [8]. These isoforms have identical N-terminal ligand binding domains but distinct intracellular C-terminal domains. With the exception of Ob-Re that lacks a transmembrane domain, the Ob-R isoforms are subdivided into short forms of the receptor (Ob-Ra, c, d and f) and a long form (Ob-Rb) with an extended intracellular region (302 residues). Ob-Rb is the predominant signaling isoform as various motifs required for the initiation of signaling are contained within its extended C-terminal domain.

## Leptin receptor expression in the CNS

In rodents and humans, leptin receptor mRNA and protein are highly expressed in hypothalamic brain regions involved in regulating energy homeostasis including the ventromedial hypothalamus, arcuate nucleus and dorsomedial hypothalamus [9,10]. However, Ob-Rs are also highly expressed in many extra-hypothalamic brain regions including the amygdala, cerebellum, hippocampus, brain stem and substantia nigra [11]. More recently genetic reporter systems have identified the brain regions that express ObRb, and in accordance with previous *in situ* hybridisation and immunocytochemical studies high levels of expression of ObRb were detected in hypothalamic and extra-hypothalamic brain regions [12].

## Leptin receptor signal transduction

The leptin receptor displays greatest homology with the class I cytokine receptor superfamily; receptors within this family lack intrinsic tyrosine kinase activity and signal via association with janus tyrosine kinases (JAKs; Ihle, 1995). Leptin binding to Ob-Rb results in the activation and phosphorylation of JAK2, which enables recruitment of various downstream signaling molecules including insulin receptor substrate (IRS) proteins, the p85 subunit of phosphoinositide 3-kinase (PI 3-kinase) and STAT (signal transducers and activators of transcription) transcription factors. Another target for JAKs is the adaptor protein Src homology collagen (Shc), which interacts with Grb2, and in turn recruits the Son-of-sevenless (Sos) exchange protein to the plasma membrane culminating in the activation of the Ras-Raf-MAPK pathway [13].

## Modulation of hypothalamic neuron excitability by leptin

Within the hypothalamus, two neuronal subtypes are key targets for the actions of leptin with respect to its effects on food intake and body weight: the orexigenic neuropeptide Y (NPY) and agouti-related protein (AGRP)-containing neurons, and anorexigenic proopiomelanocortin (POMC) and cocaine and amphetamine regulated transcript (CART)-containing neurons, respectively. Electrophysiological studies have shown that leptin hyperpolarises glucose-responsive NPY/AGRP neurons via activation of ATP-sensitive K^+^ (K_ATP_) channels [14]; an action likely to result in decreased action potential firing frequency in NPY/AGRP neurons [15], a reduction in transmitter output and ultimately reduced food intake. Opening of K_ATP_ channels is rapid as channel activation occurs within 10 min of leptin addition. Furthermore, inhibitors of PI 3-kinase prevent channel activation by leptin thereby implicating PI 3-kinase in leptin receptor coupling to K_ATP_ channels [14]. In a manner similar to the hypothalamus, leptin also rapidly inhibits neurons located within the dorsal motor nucleus of the vagus and the nucleus tractus solitarius via PI 3-kinase-driven activation of K_ATP_ channels [16,17].

It is well documented that PI 3-kinase promotes the phosphorylation of PtdIns(4,5)P_2_ into PtdIns(3,4,5)P_3_. Indeed, in a hypothalamic cell line, leptin increases PtdIns(3,4,5)P_3_ immunoreactivity [18^•^]. Moreover, genetic inactivation of PTEN in hypothalamic neurons has shown that PtdIns(3,4,5)P_3_-dependent signaling is pivotal for K_ATP_ channel activation [19^••^]. Functional inactivation of PTEN by leptin is also reported to increase PtdIns(3,4,5)P_3_ levels and promote K_ATP_ channel activation in an hypothalamic cell line [18^•^]. This not only suggests that the PTEN phosphatase is a novel component of leptin signaling but also that the excitability of hypothalamic neurons can be controlled by regulation of this phosphatase by leptin. The lipid products of PI 3-kinase closely associate with the actin cytoskeleton, and re-organisation of actin filaments plays a key role in the opening of numerous ion channels. Indeed, leptin activation of K_ATP_ channels in hypothalamic neurons [20] and insulinoma cells [21] involves PI 3-kinase-driven depolymerisation of actin filaments. However, in these cells it remains to be determined how the leptin dependent increase in PtdIns(3,4,5)P_3_ levels influences actin dynamics.

## Leptin regulation of hippocampal excitability

In contrast to its actions on hypothalamic neurons, leptin inhibits hippocampal neurons via activating large conductance Ca^2+^-activated K^+^ (BK), but not K_ATP_, channels [22]. Although the potassium channels targeted by leptin in the hippocampus and hypothalamus differ, the signaling pathway underlying their activation by leptin is analogous as a PI 3-kinase-driven signaling cascade couples leptin receptors to hippocampal BK channels [22]. In support of a PI 3-kinase-mediated event in BK channel activation, exposure of hippocampal neurons to leptin results in a rapid and highly localised elevation in the synaptic levels of PtdIns(3,4,5)P_3_. This in turn promotes re-organisation of actin filaments that ultimately results in the activation as well as the trafficking of BK channels to hippocampal synapses [23]. As hippocampal BK channels are responsible for repolarising action potentials and influence action potential firing rates, this leptin-dependent sequence of events is likely to have important implications for hippocampal excitability. Indeed, Shanley *et al.* [24] demonstrated that leptin has powerful anti-convulsant properties as demonstrated by its ability to significantly reduce hippocampal excitability in two distinct epilepsy models. The effects of leptin have also been assessed in a penicillin model of epilepsy in the somatomoter cortex. However, in this brain region leptin is reported to have pro-convulsant activity [25], suggesting that there may be regional differences in the capacity of leptin to influence neuronal hyperexcitability.

In addition to regulating hippocampal excitability by modulating BK channel function, there is evidence that leptin evokes a novel form of NMDA receptor-dependent long-term depression (LTD) under conditions of enhanced excitability [26]. The ability of leptin to markedly alter the efficacy of excitatory synaptic transmission under such conditions indicates that leptin can regulate hippocampal neuron excitability via both synaptic and non-synaptic mechanisms. However, in contrast to the PI 3-kinase-dependent events underlying leptin activation of BK channels, leptin-induced LTD is negatively regulated by PI 3-kinase and serine/threonine phosphatases 1/2A [26], indicating that distinct cellular signaling mechanisms are involved in these phenomenon.

## The effects of leptin on brain reward circuitry

Recently, leptin receptor expression has been detected on mesolimbic dopamine neurons within the basal ganglia [27]. Moreover, administration of leptin, either peripherally to anaesthetised rats or acutely to *in vitro* brain slices, results in marked attenuation of the firing frequency of VTA dopaminergic neurons [28^••^], indicating that leptin plays a role in regulating the mesolimbic dopamine system. Leptin deficiency also results in decreases in the vesicular somatodendritic stores of dopamine [29], suggesting that leptin tonically inhibits dopamine neurons within the VTA leading to a decrease in both dopamine release and food intake. Although there is evidence that leptin promotes phosphorylation of STAT3 in VTA dopaminergic neurons [28^••^,30], the precise cellular mechanisms underlying the direct effects of leptin on mesolimbic neuron excitability remain to be determined.

## Leptin influences hippocampal-dependent learning and memory

It is well established that learning and memory is a key function of the hippocampus. Indeed, one form of synaptic plasticity known as long-term potentiation (LTP) occurs in this brain region; a phenomenon thought to be a cellular correlate of certain aspects of learning, memory and habituation. In the hippocampal CA1 region, *N*-methyl-d-aspartate (NMDA) receptor-dependent LTP contributes to the formation of spatial memory [31]. Recently, several studies have implicated the hormone leptin in hippocampal synaptic plasticity. Indeed, rodents with leptin receptor mutations (*db*/*db* mice or *fa*/*fa* rats) exhibit impairments in hippocampal LTP and long-term depression [32] as well deficits in hippocampal-specific memory tasks [32,33]. Direct administration of leptin into the hippocampus improves learning and memory performance [34,35] and facilitates hippocampal LTP [36]. Furthermore, leptin facilitates the conversion of short-term potentiation (STP) into LTP [37] and enhances LTP at hippocampal CA1 synapses [35]. The synaptic activation of NMDA receptors and a concomitant postsynaptic rise in intracellular Ca^2+^ are pre-requisites for the LTP induction in the CA1 region [31]. Moreover, several studies have demonstrated that numerous growth factors and hormones can influence hippocampal LTP; actions that predominantly occur via modulation of NMDA receptor function. Similarly, leptin displays NMDA-enhancing properties as it rapidly facilitates both NMDA receptor-dependent synaptic currents in hippocampal slices from two-week-old to three-week-old rats and NMDA-induced Ca^2+^ influx in hippocampal cultures via activation of PI 3-kinase and MAPK-dependent signaling cascades [37]. Moreover, in *Xenopus* oocytes expressing recombinant NMDA receptors, leptin enhanced currents evoked by maximal, as well as submaximal concentrations of NMDA [38], suggesting that the density of functional NMDA receptors is increased by leptin. However, the ability of leptin to modulate NMDA receptor function may be age-dependent as leptin is also reported to inhibit, rather than enhance, NMDA-induced currents in hippocampal slices from six-week-old to seven-week-old rats [35].

Evidence is accumulating that structural changes play a pivotal role in activity-dependent synaptic plasticity and this process is influenced by various neurotrophins. Recently, O’Malley *et al.* [39^•^] demonstrated that leptin promotes rapid remodeling of hippocampal dendrites, via a process requiring the synaptic activation of NR2A-containing NMDA receptors and the MAPK (ERK) signaling cascade. This effect of leptin is associated with the formation of new synaptic connections as leptin also rapidly enhanced the density of hippocampal synapses. Previous studies have shown that the changes in dendritic morphology evoked by neurotrophins such as BDNF occur after several hours exposure to these agents. By contrast, the dendritic remodeling induced by leptin occurs over a much faster time scale as it is evident within a matter of minutes. Moreover, the time course of these leptin-induced changes is similar to the structural changes reported to occur following the induction of hippocampal LTP. These findings not only lend further support to the proposed role for leptin in hippocampal synaptic plasticity, but also indicate that leptin has the capacity to rapidly remodel dendrites that in turn may underlie its effects on hippocampal LTP.

## Metabolic disturbance and the development of neurodegenerative disorders

Several lines of evidence have implicated dysregulation and/or deficiencies in the leptin system in the cognitive deficits associated with neurodegenerative disorders such as Alzheimer's disease. Indeed reductions in the circulating levels of leptin have been detected in Alzheimer's disease patients [40]. Moreover, leptin is reported to significantly decrease the levels of amyloid β [41] and to improve memory performance [34] in transgenic mouse models of Alzheimer's disease. It is known that obesity in humans closely correlates with the development of type II diabetes. Moreover, cognitive deficits are prevalent in diabetic patients. As obesity and obesity-linked disorders such as type II diabetes are associated with leptin resistance at the level of the blood brain barrier [42], it is likely that CNS insensitivity to leptin plays a role in the cognitive impairments in these individuals.

## Leptin is a novel anti-depressant

In addition to its role in hippocampal-dependent learning and memory, recent studies have demonstrated that leptin has anti-depressant-like activity. Indeed, exposure of rodents to stress paradigms, which induce various behavioural deficits resembling certain aspects of human depression, results in significant decreases in the circulating levels of leptin [43^•^]. Moreover, systemic administration of leptin reversed the impairments in rewarding behaviour that occurs in chronically stressed rats; an effect that mirrors the actions of anti-depressant drugs in similar animal models. Hippocampal leptin receptors are likely to be the site of leptin's anti-depressant actions as administration of leptin into the hippocampus, but not hypothalamus, evoked anti-depressant-like effects in the forced swim test. Thus, these data suggest that leptin is a novel anti-depressant and that dysregulation of the leptin system may be an underlying feature of certain depressive disorders. This raises the interesting possibility that agents that enhance leptin receptor-driven signaling may be useful therapeutic targets in the treatment of depression.

## Conclusions

Evidence is accumulating that leptin plays a pivotal role in modulating diverse neuronal functions. Recent studies have shown that leptin rapidly regulates the excitability of neurons via potassium channel activation in a number of brain regions. In addition, transgenic studies combined with advanced molecular approaches have greatly progressed our understanding of the complex cellular mechanisms coupling leptin receptors to potassium channel activation as well as the resulting functional outputs. In other studies, a role for leptin in modulating hippocampal-dependent cognitive function has been identified. Moreover, major advances have been made in the characterisation of the cellular targets, signaling pathways and structural changes that underlie the effects of leptin on these hippocampal-driven processes.

Although the cellular consequences of leptin receptor activation are becoming well characterised, there are still gaps in our knowledge of how the neuronal leptin system functions. For instance, the conditions under which physiologically relevant concentrations of leptin reach the extra-hypothalamic brain regions that respond to this hormone are not clear. Although there is evidence for leptin mRNA in the brain, it is not known if leptin can be released from neurons, and if so the processes regulating the release mechanisms. Many studies have demonstrated that leptin is an important modulator of activity-dependent synaptic plasticity and neuronal development. Moreover, it is well known that developmental processes are under the control of various growth factors and neurotrophins. However, it is not clear if and how the effects of leptin on synaptic plasticity and neurodevelopment are influenced by other hormones and growth factors.

Another important focus of current research is the association between obesity-linked leptin resistance and the development of neurodegenerative disorders such as Alzheimer's disease. This, combined with the recent discovery of the anti-depressant properties of leptin, may ultimately result in the generation of promising new therapies to treat a wide spectrum of neurological conditions.

## References and recommended reading

Papers of particular interest, published within the annual period of review, have been highlighted as:

• of special interest
•• of outstanding interest
